# Glucocorticoid receptor gene polymorphisms and susceptibility to rheumatoid arthritis

**DOI:** 10.1111/j.1365-2265.2007.02887.x

**Published:** 2007-09

**Authors:** Rachelle Donn, Debbie Payne, David Ray

**Affiliations:** * Arthritis Research Campaign Epidemiology Unit (ARC/EU) Manchester, UK; † Centre for Molecular Medicine (CMM) Manchester, UK; ‡ Centre for Integrated Genomic Medical Research, University of Manchester Manchester, UK

## Abstract

**Background:**

A defect in hypothalamic-pituitary-adrenal (HPA) axis function has been suggested to contribute to susceptibility to rheumatoid arthritis (RA).

**Objective:**

To investigate polymorphisms of the glucocorticoid receptor (GR) gene and determine any associations with RA.

**Methods:**

Three GR polymorphisms that tag 95% of all haplotypes across the GR gene were genotyped. These are an intron B Bcl1 polymorphism, a ttg insertion/deletion within intron F (rs2307674) and the single nucleotide polymorphism (SNP) lying in the 3′ untranslated region of exon 9b (rs6198). The dye terminator-based SNaPshot method or size resolution by capillary electrophoresis was performed. The study population comprised 198 UK Caucasian RA cases and 393 ethnically matched controls.

**Results:**

No significant single point or haplotypic associations were found for GR polymorphisms with RA susceptibility. Furthermore, no evidence for GR polymorphisms with aspects of RA severity was seen.

**Conclusion:**

In this study of the most comprehensive coverage of GR polymorphisms with RA, no significant contributing role for GR polymorphisms with RA was found.

## Introduction

Studies from both animals and humans suggest that a defect in the neuroendocrine system may be important in the disease rheumatoid arthritis (RA). Inappropriately normal plasma cortisol and a blunted response of plasma cortisol to surgical stress have been shown for RA patients.^1^,^2^ Glucocorticoids (Gcs) are required for the development of streptococcal cell wall-induced arthritis in Lewis rats.^3^ Furthermore, while many RA patients respond well to exogenous Gc treatment, a proportion fail to do so.^4^ These observations suggest that hypothalamic-pituitary-adrenal (HPA) axis dysregulation or relative Gc deficiency contributes to the onset of RA.

Gcs exert their effect by binding to the intracellular receptor, the glucocorticoid receptor alpha (GRα). The human GR gene (GCCR, GCR, GRL, NR3C1) (locus 5q31) is 10 exons in length and exons 1-9α are transcribed into GRα mRNA, which is translated into a functional receptor (GRα). Alternative splicing of the primary transcript results in an mRNA containing exons 1-9β, which gives rise to the GRβ. Unlike GRα, GRβ does not bind hormone and is transcriptionally inactive.^5^,^6^ Although a dominant negative effect of GRβ on GRα activity has been known for some time, the mechanism by which this occurs is not known and the precise functional significance of GRβ remains unclear. Several associations with GRβ overexpression and autoimmune/inflammatory diseases have been described. It is not known, however, whether this is a cause or consequence of the inflammatory state and whether the increase in the GRβ is sufficient to exert a dominant negative effect on GRα. It has been suggested that GRβ could in part mediate Gc resistance seen in RA.^7^

Derijk *et al*. described an A to G polymorphism at position 3736 in exon 9b (now rs6198).^8^ This polymorphism lies in an AUUUA motif of the 3′ UTR of the mRNA of the GRβ isoform and has been shown to increase mRNA stability and also receptor protein expression.^9^

**Fig. 1:**
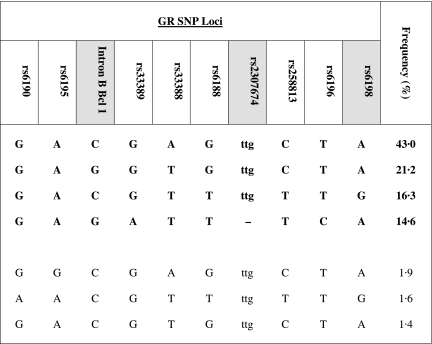
Haplotype tagging SNPs. Haplotypes (*n* = 425) occurring with a frequency of > 1% are shown. Boldface indicates the haplotypes captured by the three haplotype tagging polymorphisms (shaded): intron B Bcl1, C → G, the presence or absence of the ttg insertion (rs2307674) and the rs6198 A → G. (Adapted from Stevens *et al.*^10^)

Derijk *et al*. originally detailed the genotype and allele frequencies of the rs6198 polymorphism in a very small sample size of 30 RA cases compared with 24 controls and found carriage of the mutant allele to be associated with significant increased risk of RA.^8^ Here we report our attempt to replicate and extend these findings. We have previously demonstrated strong linkage disequilibrium across the GR gene locus and suggested haplotype tagging single nucleotide polymorphisms (SNPs).^10^ In this current study we looked at three polymorphisms that in combination capture 95% of all haplotypes occurring at a frequency of greater than 1% in Caucasians (Fig. 1). These are an intron B Bcl1 polymorphism, which has been associated with increased Gc sensitivity and a lower body mass index,^11^ a ttg insertion/deletion within intron F (rs2307674) and the SNP lying in the 3′ untranslated region of exon 9b (rs6198). We studied these in a well-characterized panel of RA patients and healthy controls to determine whether polymorphisms of GR contribute to RA susceptibility. Utilizing this combination of SNPs allows for the most comprehensive investigation of GR polymorphisms in RA that has been carried out to date.

## Methods

### Patients and controls

Blood samples were obtained with informed written consent. Ethical approval was obtained from Multicentre Research Ethics Committee (MREC) (99/8/84) and the University of Manchester committee on the ethics of research on human beings (8/92/(i)).

All patients and controls were of UK Caucasoid origin.

One hundred and ninety-eight RA cases from the Arthritis Research Campaign National Repository for families with RA were studied. Only one affected case per family was selected, at random, for investigation. All RA cases had disease that met the American College of Rheumatology 1987 criteria, as modified for genetic studies.

DNA was available for 393 control subjects. These were individuals recruited from blood donors and from the records of general practitioners.

### Genotyping

SNP genotyping was by the dye terminator-based SNaPshot method (Applied Biosystems, Warrington, UK). In brief, polymerase chain reactions (10 µl final volume) contained 10 ng of genomic DNA, 0·4 µl of each primer (25 pmol/µl), 1 µl of 10 × NH_4_ buffer (Bioline), 1 µl of dNTPs (Bioline, 2 mM), 0·3 µl MgCl_2_ (Bioline, 1·5 mM) and 0·2 µl of Taq polymerase (Bioline, 5 U/µl). The primers and probes used were (5′ to 3′): for the intron B Bcl1 SNP: forward primer CTA TTC TTC AAA CTG AAT CTT CTG, reverse primer CAA CAC GTA TAT CTA CAT TTA GAA C, and probe GAC ACC AAT TCC TCT CTT AAA GAG ATT; for the rs6198 SNP: forward primer AGT GTC TTT TTA CCT ACG C, reverse primer ATG TTT CTC CAT ATT TGG C, and probe TGT GGT TTG GTA ATA CCA GAA CAG CAA ATT TAA A.

The presence or absence of a ttg insert (rs2307674) was determined using capillary electrophoresis for size resolution, relative to a ROX 450 internal size standard, on an ABI Prism 310 DNA Genetic Analyser (Applied Biosystems), using a fluorescent dye (HEX)-labelled forward primer. The results were analysed using Genescan analysis and Genotyper 3·6 software (Applied Biosystems). The rs2307674 forward primer was ACC TCA AGT GAT CCA CCC, and the reverse primer GTA CAT GGT TAT ACT CAT ATA TAA C.

The RA cases and a proportion of the controls had been previously genotyped for HLA-DRB1 alleles and shared epitope (SE) status assigned.

### Statistical analysis

Associations between the GR polymorphisms and RA were analysed using the χ^2^-test (Stata, College Station, TX) and correction for multiple testing was performed by Bonferroni's method. Haplotype analysis was carried out using the expectation-maximization algorithm implemented in HelixTree (Golden Helix, Bozeman, MT). Stratification analysis was performed to investigate the effects of gender, disease severity, as assessed by the presence of erosive disease, age at disease onset (the median age at onset was used as the cut-off value), and carriage of SE alleles (HLA-DRB1*0401, *0404, *0101, *0102, *1001 and *1402 were considered positive for the shared epitope).

The study had > 85% power to detect an odds ratio (OR) of 1·8 or greater with RA susceptibility (*P =* 0·05).

## Results

No deviation from Hardy-Weinberg equilibrium was seen for any polymorphism in the cases or the controls. Strong linkage disequilibrium (LD) (*r*^2^ = 1) was seen for the polymorphisms studied in both the case and control groups.

No association with any GR polymorphism was found with the RA cases when considered as a whole group (Table 1). Similarly, no significant haplotype associations were seen with RA susceptibility.

**Table 1:** Frequency of intron B Bcl1, rs2307674 and rs6198 GR SNP genotypes in control and RA cases

GR polymorphism	Controls *n* (%)	RA cases *n* (%)	*P*-value
Intron B BCl1	(*n* = 392)	(*n* = 195)	
CC	147 (37·5)	76 (39·0)	
CG	195 (49·7)	94 (48·2)	
GG	50 (12·8)	25 (12·8)	0·81
	(*n* = 384)	(*n* = 191)	
rs2307674			
ttg/ttg	255 (66·4)	136 (71·2)	
ttg/-	119 (31·0)	47 (24·6)	
-/-	10 (2·6)	8 (4·2)	0·50
	(*n* = 390)	(*n* = 178)	
rs6198			
AA	256 (65·6)	129 (72·5)	
AG	121 (31·1)	44 (24·7)	
GG	13 (3·3)	5 (2·8)	0·13

On stratified analysis, a weak positive association with an excess of rs6198 AA (wild-type) homozygotes was seen when the SE negative cases were compared with SE negative controls (Table 2). Furthermore, use of Bonferroni's correction for multiple testing (*n* = 3) rendered this observation nonsignificant (*P*_corr_ = 0·15).

**Table 2:** Frequency of intron B BCl1, rs2307674 and rs6198 GR SNP genotypes in controls and in the RA cohort stratified by erosive disease, age at disease onset, and carriage of SE alleles

						SE status			
		Erosive disease		Age at disease onset		SE negative		SE positive	
Genotype	Controls	Present	Absent	< 41 years	≥ 42 years	Controls	RA cases	Controls	RA cases
Intron B BCl1	(*n* = 392)	(*n* = 139)	(*n* = 25)	(*n* = 75)	(*n* = 72)	(*n* = 71)	(*n* = 41)	(*n* = 86)	(*n* = 154)
CC	147 (37·5)	58 (41·8)	7 (28·0)	32 (42·7)	31 (43·1)	29 (40·8)	16 (39·1)	29 (33·7)	60 (39·0)
CG	195 (49·7)	63 (45·3)	14 (56·0)	30 (40·0)	28 (38·9)	35 (49·3)	19 (46·3)	47 (54·7)	75 (48·7)
GG	50 (12·8)	18 (12·9)	4 (16·0)	13 (17·3)	13 (18·0)	7 (9·9)	6 (14·6)	10 (11·6)	19 (12·3)
*P*-value	–	0·55	0·37	0·95	0·98	–	0·62	–	0·62
Rs2307674	(*n* = 384)	(*n* = 135)	(*n* = 25)	(*n* = 73)	(*n* = 72)	(*n* = 71)	(*n* = 41)	(n-86)	(*n* = 150)
ttg/ttg	255 (66·4)	101 (74·8)	15 (60·0)	57 (78·1)	31 (43·1)	51 (71·8)	27 (65·9)	56 (65·1)	109 (72·7)
ttg/–	119 (31·0)	31 (23·0)	7 (28·0)	10 (13·7)	28 (38·9)	20 (28·2)	11 (26·8)	26 (30·2)	36 (24·0)
–/–	10 (2·6)	3 (2·2)	3 (12·0)	6 (8·2)	13 (18·0)	0 (0·0)	3 (7·3)	4 (4·7)	5 (3·3)
*P*-value	–	0·10	0·16	0·38	0·38	–	0·20	–	0·22
rs6198	(*n* = 390)	(*n* = 123)	(*n* = 23)	(*n* = 66)	(*n* = 63)	(*n* = 70)	(*n* = 37)	(*n* = 87)	(*n* = 141)
AA	256 (65·6)	87 (70·7)	19 (82·6)	45 (68·2)	41 (65·0)	44 (62·9)	30 (81·1)	56 (64·4)	99 (70·2)
AG	121 (31·1)	31 (25·2)	4 (17·4)	18 (27·3)	19 (30·2)	22 (31·4)	6 (16·2)	27 (31·0)	38 (27·0)
GG	13 (3·3)	5 (4·1)	0 (0·0)	3 (4·5)	3 (4·8)	4 (5·7)	1 (2·7)	4 (4·6)	4 (2·8)
*P*-value	–	0·44	0·08	0·86	0·79	–	0·05	–	0·30

No other single or haplotypic association of GR polymorphisms and no other parameters of RA severity were found.

## Discussion

Inappropriately low endogenous cortisol production may be a contributing factor to the onset and progression of RA. How this arises has not been ascertained.^12^ Alternatively, a normal HPA axis response to inflammation may be compromised in RA patients by a reduction or alteration in GR function. The number of GRα in lymphocytes from RA cases was found to be lower than in controls.^13^ However, the serum cortisol levels were normal so such apparent downregulation of receptor expression is difficult to explain. Polymorphism of the GR gene may influence GC sensitivity. Certain low-frequency polymorphisms of GR (ER22/23EK) have been found to be associated with parameters of Gc sensitivity in normal individuals.^14^ These nucleotide changes are in strong LD with the intron B Bcl1 polymorphism in particular, and as such we find no evidence of association of them with RA susceptibility. The possibility of a GR polymorphism conferring risk for RA was raised by Derijk *et al*.^8^ This was of particular interest because the nucleotide change occurred in the 3′ UTR of the GRβ molecule. If RA cases had an excessive amount of this variation, which was subsequently found to have functional significance and affect GRβ mRNA stability, then a possible mechanism for relative GR resistance would have been found. Our study aimed to look for variation in GR polymorphism with RA susceptibility. We specifically genotyped for the rs6198 SNP within the 3′ UTR of GRβ, in combination with two other changes, which gave excellent genetic coverage across the GR gene locus. We have accounted for 95% of all haplotypes across the GR gene by using this approach.

No evidence for genetic association with GR polymorphisms and RA susceptibility was observed. This is in agreement with the findings of a Korean study of RA, in which the 149 RA cases and controls were compared and no association with the intron B Bcl1 polymorphism was found with the RA group as a whole, or on stratification for disease severity.^15^ Although less well powered to look at, we have also stratified our patient cohort by parameters of disease severity. An excess of rs6198 AA (wild-type) homozygotes was seen when the SE negative cases were compared with the SE negative controls (Table 2). However, given the relatively small numbers involved, and the borderline level of significance, which is not maintained once correction for multiple testing is applied, this finding needs to be interpreted with caution.

In summary, we have looked at three changes across the GR gene that capture 95% of all commonly occurring haplotypes, but find no evidence for GR polymorphisms contributing a substantial genetic effect, either as single point or as haplotypic associations, to risk of RA. We conclude that GR polymorphisms do not confer significant risk of RA.
